# Zika Virus Is Not Uniquely Stable at Physiological Temperatures Compared to Other Flaviviruses

**DOI:** 10.1128/mBio.01396-16

**Published:** 2016-09-06

**Authors:** Leslie Goo, Kimberly A. Dowd, Alexander R. Y. Smith, Rebecca S. Pelc, Christina R. DeMaso, Theodore C. Pierson

**Affiliations:** Viral Pathogenesis Section, National Institute of Allergy and Infectious Diseases, National Institutes of Health, Bethesda, Maryland, USA

## Abstract

Zika virus (ZIKV) is a flavivirus that has emerged as a global health threat due in part to its association with congenital abnormalities. Other globally relevant flaviviruses include dengue virus (DENV) and West Nile virus (WNV). High-resolution structures of ZIKV reveal many similarities to DENV and suggest some differences, including an extended glycan loop (D. Sirohi, Z. Chen, L. Sun, T. Klose, T. C. Pierson, et al., 352:467–470, 2016, http://dx.doi.org/10.1126/science.aaf5316) and unique interactions among envelope (E) protein residues that were proposed to confer increased virion stability and contribute mechanistically to the distinctive pathobiology of ZIKV (V. A. Kostyuchenko, E. X. Lim, S. Zhang, G. Fibriansah, T. S. Ng, et al., Nature 533:425–428, 2016, http://dx.doi.org/10.1038/nature17994). However, in the latter study, virus stability was inferred by measuring the loss of infectivity following a short incubation period. Here, we rigorously assessed the relative stability of ZIKV, DENV, and WNV by measuring changes in infectivity following prolonged incubation at physiological temperatures. At 37°C, the half-life of ZIKV was approximately twice as long as the half-life of DENV (11.8 and 5.2 h, respectively) but shorter than that of WNV (17.7 h). Incubation at 40°C accelerated the loss of ZIKV infectivity. Increasing virion maturation efficiency modestly increased ZIKV stability, as observed previously with WNV and DENV. Finally, mutations at E residues predicted to confer increased stability to ZIKV did not affect virion half-life. Our results demonstrate that ZIKV is not uniquely stable relative to other flaviviruses, suggesting that its unique pathobiology is explained by an alternative mechanism.

## IMPORTANCE

Zika virus (ZIKV) belongs to the *Flavivirus* genus, which includes other clinically relevant mosquito-borne pathogens such as dengue virus (DENV) and West Nile virus (WNV). Historically, ZIKV infection was characterized by a self-limiting, mild disease, but recent outbreaks have been associated with severe clinical complications, including Guillain-Barré syndrome and microcephaly, which are atypical of other flavivirus infections. Moreover, ZIKV has been detected in saliva, urine, and semen, and it may be sexually transmitted. Analysis of a high-resolution cryo-electron microscopic reconstruction of ZIKV hypothesized that the unusual stability of this virus contributes to its distinctive pathobiology. Here, we directly compared the stability of ZIKV to that of other flaviviruses following prolonged incubation in solution at physiological temperatures. We found that the stability of multiple ZIKV strains, including those from recent outbreaks, is intermediate between that of DENV and WNV, suggesting an alternative explanation for the unique clinical manifestations of ZIKV infection.

## Observation

Zika virus (ZIKV) is a flavivirus transmitted by the *Aedes* species of mosquitoes. ZIKV was first isolated from a rhesus macaque in 1947 in the Zika forest of Uganda (1). Documented cases of ZIKV infection of humans were relatively rare during the next five decades, despite serological studies suggesting widespread exposure (2). ZIKV began to receive global attention in 2007, when it caused a series of epidemics across islands in the Pacific Ocean (3, 4). In 2014 to 2015, ZIKV was introduced into Brazil (5, 6), and it has rapidly spread throughout the Americas (2).

Historically, ZIKV infection was characterized by a mild self-limiting febrile illness and maculopapular rash, but recent ZIKV outbreaks have been associated with severe disease, including congenital microcephaly of infants born to infected pregnant women (7, 8) and Guillain-Barré syndrome in adults (9). These clinical presentations are not typically associated with other flavivirus infections. Furthermore, ZIKV has been detected in urine (10), saliva (11), and semen (12), and it may be sexually transmitted (13, 14). The mechanism(s) for the unique pathogenesis and apparent alternative transmission routes of ZIKV is unknown. A recent study suggested that ZIKV particles displayed much greater structural stability than DENV, leading to the hypothesis that the thermal stability of ZIKV might allow it to withstand the harsh conditions of bodily fluids (15). However, virus stability in that study was inferred by measuring the loss of infectivity following a short incubation period (30 to 60 min) at 37°C or 40°C, which does not measure the true rate of decay of infectivity. Indeed, the ~30-min half-life of DENV suggested by this method differed considerably from those reported by previous studies (16–19).

**FIG 1:**
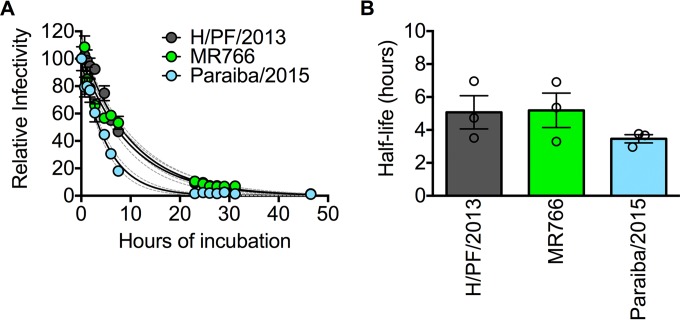
Stability of multiple strains of infectious ZIKV. (A) Representative decay curves for ZIKV strains H/PF/2013, MR766, and Paraiba/2015. Viruses were equilibrated at 37°C for 1 h (reference time point), followed by further incubation for additional lengths of time as indicated on the *x* axis, after which aliquots were harvested and frozen. Samples from each time point were concurrently thawed and used to infect Raji-DC-SIGN-R cells. Data were normalized to the infectivity observed at the reference time point and fitted to a one-phase exponential decay curve. Values are means ± standard errors of the means (error bars) from triplicate infections. Dashed lines represent the 95% confidence intervals of the regression analysis. (B) Average half-life values of ZIKV strains shown in panel A obtained from three independent experiments. Error bars indicate standard errors of the means.

**FIG 2:**
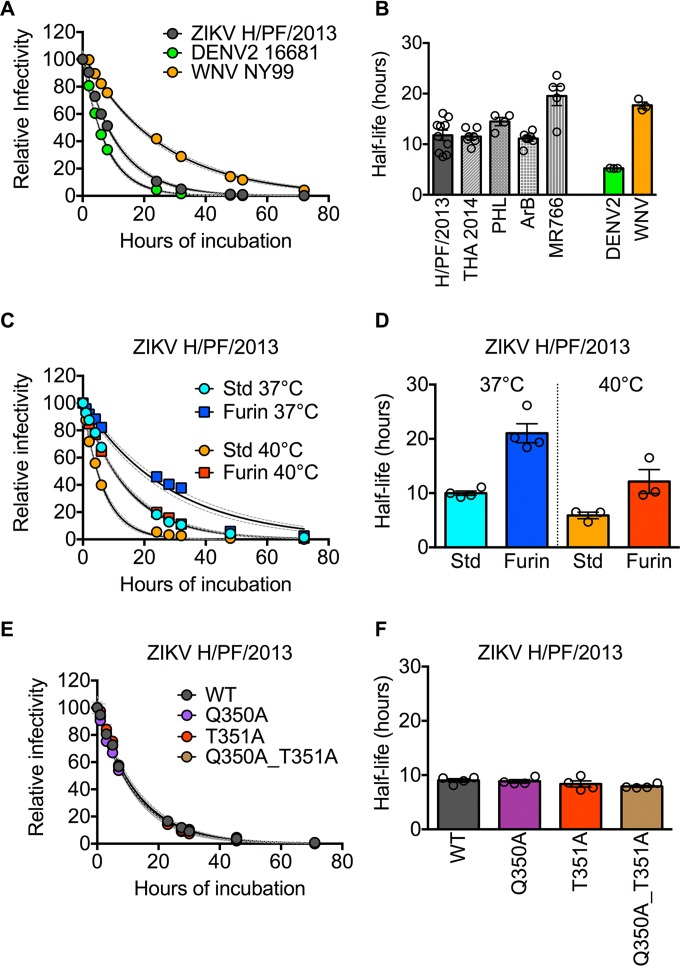
Comparative stability of flavivirus RVPs. (A) Representative decay curves for ZIKV H/PF/2013, DENV2 16681, and WNV NY99 RVPs. (B) Average half-life values of ZIKV strains compared to those of DENV2 and WNV obtained from 3 to 10 independent experiments performed in triplicate. Error bars indicate the standard errors of the means. (C) Representative decay curves for ZIKV H/PF/2013 RVPs prepared using standard conditions (Std) or in the presence of ectopically expressed human furin (Furin) to increase the efficiency of virion maturation. (D) Average half-life values of Std and Furin ZIKV H/PF/2013 RVPs incubated at 37°C or 40°C obtained from three (40°C) or four (37°C) independent experiments performed in triplicate. Error bars indicate the standard errors of the means. (E) Representative decay curves comparing wild-type (WT) or mutant ZIKV H/PF2013 RVPs. (F) Average half-life values of WT and mutant ZIKV H/PF/2013 RVPs were obtained from four independent experiments performed in triplicate. Error bars indicate the standard errors of the means. For panels A, C, and E, error bars, where visible, indicate the standard errors of the means from triplicate infections. Dashed lines represent the 95% confidence intervals of the regression analysis. All experiments were performed as in Fig. 1, except viruses were incubated at 37°C or 40°C as indicated in panels C and D.

We sought to quantitatively investigate the stability of ZIKV compared to other flaviviruses following prolonged incubation in solution at physiological temperatures. Infectious ZIKV particles representing contemporary (H/PF/2013 and Paraiba/2015) and historic (MR766) strains were incubated at 37°C and sampled periodically for up to 48 h, after which infectivity at each time point was determined simultaneously. By fitting the resulting data points (10 to 15 time points for each virus) with a one-phase exponential decay model, we obtained an average half-life of 5.1, 5.2, and 3.5 h for strains H/PF/2013, MR766, and Paraiba/2015, respectively (*n* = 3) (Fig. 1A and B). To facilitate direct comparisons with other flaviviruses, such as dengue virus type 2 (DENV2) and WNV, as well as with previous studies (16–19), we generated pseudoinfectious reporter virus particles (RVPs) (20) expressing the structural proteins of ZIKV strain H/PF/2013, for which high-resolution cryo-electron microscopy structures have been described (15, 21). In agreement with previous findings (17, 22), the average half-lives of DENV2 and WNV RVPs following prolonged incubation at 37°C were 5.2 and 17.7 h, respectively (*n* = 3) (Fig. 2A and B). The rate of decay of ZIKV H/PF/2013 RVPs (average half-life of 11.8 h) was slower than the rate of decay of DENV2 (2.3-fold difference; *P* = 0.006) but faster than that of WNV (1.5-fold difference; *P* = 0.02). To extend our findings, we also created RVPs that incorporate the structural proteins of additional ZIKV strains representing both African (MR766 and ArB770) and Asian (PHL/2012 and THA/2014) lineages. These four additional ZIKV RVP strains had an average half-life of 14.2 h (range, 11.1 to 19.6 h), with the African MR766 strain displaying a modest increase in stability relative to the other strains (1.4- to 1.7-fold increase in half-life [*P* < 0.0001]; Fig. 2B). Together, these results demonstrate that the stability of ZIKV is more comparable to other flaviviruses than previously suggested (15). Moreover, because the historic ZIKV MR766 strain displayed greater stability than contemporary strains such as Paraiba/2015, which was isolated from the 2015 epidemic in Brazil, it is unlikely that the unique clinical manifestations associated with recent epidemics are explained by increased virion stability.

Flaviviruses are structurally heterogeneous due to an inefficient maturation process responsible for cleaving a chaperone protein, prM, on the virion surface (23). As a result, flaviviruses released from infected cells may contain different amounts of uncleaved prM. Because the presence of uncleaved prM on virions modulates stability (18), we next compared the rate of decay of ZIKV RVPs produced under standard (Std) conditions or in the presence of overexpressed human furin (Furin) to increase maturation efficiency (18). As previously shown for WNV and DENV (18), increasing ZIKV maturation efficiency resulted in a slower rate of decay of infectivity: the stability of Furin ZIKV RVPs was approximately twofold greater than Std ZIKV RVPs at 37°C (average half-life of 21 and 10 h, respectively; Fig. 2C and D). As expected, virion stability was also modulated by temperature: prolonged incubation at 40°C reduced the infectious half-life of both Std and Furin RVPs (average half-life of 6 and 12 h, respectively; Fig. 2C and D) relative to incubation at 37°C. While we have recently shown that infectious ZIKV and ZIKV RVPs have similar antigenic structures (24), here we noted that ZIKV H/PF/2013 RVPs (produced in mammalian cells) have a slightly longer half-life than fully infectious viruses (produced in mosquito cells) (Fig. 1 and 2), which may be explained by differences in prM processing by these producer cells, as previously described (25). These findings suggest that heterogeneity in virion maturation state contributes to differences in stability among ZIKV strains and flaviviruses, as well as among virus preparations.

Prior structural analysis of ZIKV identified an insertion in E-protein domain III, which was hypothesized to promote hydrogen bond interactions between residues Q350 and T351 that increase the overall stability of ZIKV (15). To evaluate the functional contribution of these residues to ZIKV stability, we generated ZIKV RVP variants in which Q350 and T351 were replaced with alanine residues individually or in combination. Introduction of these mutations individually or in combination had no effect on ZIKV stability (maximum of 1.1-fold difference in half-life compared to wild-type [WT] RVPs [*P* = 0.23]; Fig. 2E and F).

### Conclusions.

Recent ZIKV outbreaks have been associated with unexpectedly severe clinical complications, including congenital abnormalities, that are not characteristic of other flavivirus infections. The molecular basis for the distinctive pathobiology of ZIKV is unknown. A recent analysis of a high-resolution cryo-electron microscopic reconstruction of ZIKV led to the hypothesis that the unusual stability of this virus may contribute to its unique pathobiology and routes of transmission (15). One important limitation of the functional studies used to support this conclusion was that the short interval over which virus stability was measured is suitable for capturing the biology of only an exceptionally unstable virus particle. Here, we measured the stability of viruses at physiological temperatures throughout a prolonged incubation period shown previously to be sufficient for measuring virus stability with precision (17–19, 22). Our studies with multiple strains of infectious ZIKV and ZIKV RVPs suggest that the stability of ZIKV (half-life of 11.8 h) is comparable to the stabilities of both DENV (half-life of 5.2 h) and WNV (half-life of 17.7 h). Mutation at ZIKV residues predicted to govern stability via unique interactions among five neighboring E-protein domain IIIs (15) had no measurable impact on ZIKV stability. As expected from prior studies, the stability of virions is modulated by the efficiency of virion maturation (18), which may vary among virus species, strains, and preparations. Further studies will be required to identify additional factors that modulate virion stability, including sequence variation, interactions with host factors, and environmental composition and pH. Overall, our findings demonstrate that ZIKV is not distinctly stable compared to other flaviviruses, suggesting an alternative explanation for its unique pathobiology.

### Cell lines and viruses.

HEK-293T and Vero cells were maintained in Dulbecco’s modified Eagle medium (DMEM) containing 25 mM HEPES (Invitrogen) supplemented with 7% fetal bovine serum (FBS) (Invitrogen) and 100 U/ml penicillin-streptomycin (P/S) (Invitrogen). Raji-DC-SIGN-R cells were cultured in RPMI 1640 medium containing GlutaMAX supplemented with 7% FBS and 100 U/ml P/S. HEK-293T, Vero, and Raji-DC-SIGN-R cells were maintained at 37°C in the presence of 7% CO_2_. *Aedes albopictus* C6/36 cells were grown in Eagle’s minimum essential medium (Invitrogen) supplemented with 10% FBS, 1× GlutaMAX (Invitrogen), and 1× nonessential amino acids (Invitrogen) and maintained at 30°C in the presence of 7% CO_2_. Infectious ZIKV stocks were produced by infecting C6/36 cells, as described elsewhere (24). Virus supernatant collected on days 3 to 6 was clarified, passed through a 0.22-µm filter, and stored at −80°C.

### Structural gene plasmids.

The structural genes from ZIKV strains MR766 (GenBank accession no. HQ234498), ArB7701 (GenBank accession no. KF268950), PHL/2012 (GenBank accession no. KU681082), and THA/2014 (GenBank accession no. KU681081) were synthesized and cloned into pcDNA3.1 (BioBasic and GenScript). Sequence carrying the structural genes of ZIKV strain H/PF/2013 (GenBank accession no. KJ776791) was cloned from viral stocks using standard molecular cloning techniques as previously described (24). Plasmids encoding the structural genes from the WNV lineage 1 NY99 strain (20) and from DENV2 strain 16681 (17) have been described previously.

### RVP production.

Reporter virus particles (RVPs) were produced by complementation of a previously described green fluorescent protein (GFP)-expressing WNV subgenomic replicon (20) with plasmids carrying the structural genes (capsid, prM, and E) of ZIKV, WNV, or DENV2. HEK-293T cells were preplated on a low-glucose (1-g/liter) formulation of DMEM containing 25 mM HEPES (Invitrogen), 7% FBS, and 100 U/ml P/S, transfected with plasmids carrying the replicon and structural genes at a 1:3 ratio (by mass) using Lipofectamine 3000 (Invitrogen), and incubated at 30°C. To increase the efficiency of virion maturation, a plasmid expressing human furin was included as previously described (18). For each microgram of DNA, 2 µl of Lipofectamine 3000 was used. Virus-containing supernatant was harvested on days 3 to 5 posttransfection, passed through a 0.22-µm filter (Millipore, Billerica, MA), and stored at −80°C.

### Determination of virus titer.

Clarified RVP-containing supernatant was serially diluted twofold in a total volume of 100 µl and used to infect 5 × 10^4^ Raji-DC-SIGN-R cells in an equal volume at 37°C. Cells were fixed in 1.8% paraformaldehyde at 48 h following infection, and GFP-positive cells were enumerated using flow cytometry. The infectious titer of fully infectious ZIKV was determined by intracellular staining of Raji-DC-SIGN-R cells (1 × 10^5^ in 100 µl/well) as previously described (24).

### Intrinsic decay.

Viruses were diluted to 10% infectivity in low-glucose (1-g/liter) DMEM supplemented with 7% FBS and 100 U/ml P/S, allowed to equilibrate at 37°C or 40°C for 1 h (reference), and sampled periodically for the next 48 to 72 h. At each time point, aliquots were collected and stored at −80°C. All frozen samples were thawed simultaneously and used to infect Raji-DC-SIGN-R cells in triplicate to assess infectivity as described above. Infection was normalized to the level observed at the reference time point and fitted with a one-phase exponential decay curve (GraphPad Prism v 6.0 g, GraphPad Software Inc.) to estimate the infectious half-life.

### Statistical analysis.

Mean half-life values were compared using ordinary one-way ANOVA followed by Tukey’s multiple-comparison test. Statistical analyses were performed using GraphPad Prism v 6.0 g (GraphPad Software Inc.).
